# Keeping Our ACT Team Together: Supervisory Strategies to Promote Workforce Retention

**DOI:** 10.1007/s10488-025-01458-7

**Published:** 2025-06-29

**Authors:** Mimi Choy-Brown, William Carlson, Nathaniel J. Williams, Lynette Studer, Sheetal Digari

**Affiliations:** 1 University of Minnesota– Twin Cities, St. Paul, MN USA; 2 Boise State University, Idaho, USA; 3 University of Wisconsin– Madison, Madison, WI USA

**Keywords:** Clinical supervision, Assertive community treatment, Intention to leave, Leadership

## Abstract

Strategies are urgently needed to address the mental health workforce crisis in the United States that threatens essential care for people living with psychiatric disabilities. Assertive Community Treatment (ACT) is a cornerstone of community mental health care and workforce turnover negatively impacts patient experiences and outcomes. The present study aims to examine malleable supervisory strategies for reducing the intention to leave among the ACT workforce in order to reduce voluntary turnover. A cross-sectional observational survey of the largest sample yet of ACT team members (*N* = 334) from 80 ACT teams working in four States assessed team-level and team members’ supervision characteristics theorized to predict supervisory working relationships and team members’ intention to leave their positions. Unadjusted and adjusted hierarchical linear regression models examined factors at multiple levels (i.e., team member, team) in relation to team members’ intention to leave and accounted for the clustering of team members within teams. Adjusted models indicated that team member and team factors were negatively associated with team members’ intention to leave. Specifically, higher levels of transformational leadership and strong supervisory working alliances had a significant association with reduced intention to leave. Additionally, empirically supported supervision strategies and transformational leadership were significantly associated with stronger supervisory working alliances. Strategic workforce investment in the development of ACT team leaders and their use of transformational leadership and empirically supported supervision strategies may represent a promising pathway to promote strong supervisory working alliances and retention of essential ACT team members.

## Introduction

The public mental health care workforce crisis in the United States has been widely established (Counts, 2023; Hoge & Paris, 2018), and has broad implications for the accessibility and quality of care (CDC, 2017; Cunningham & Dixon, 2020). Improving work life has been added as a fourth critical target for optimizing healthcare systems, which previously included improving population health and patient experiences while reducing costs (Bodenheimer & Sinsky, 2014). This workforce target is especially relevant to services for people with serious mental illness (CDC, 2017; Olfson, 2016). Mental health workforce turnover rates are notably high and have ranged from 30 to 60% (Beidas et al., 2016; Substance Abuse and Mental Health Services Administration, 2020). This is higher than the 20–30% of recorded staff losses during the COVID-19 pandemic in all direct health care roles (Boston-Fleischhauer, 2022; Poindexter, 2022). Some level of turnover may be beneficial for individual growth and overall system health. However, substantial human resource losses lead to significant deficits in program quality (Mancini et al., 2009), institutional knowledge, mentored and peer support (Sheather & Slattery, 2021), financial stress, and decreased fidelity to evidence-based practices (Pascoe et al., 2021).

The COVID-19 pandemic exacerbated challenges in mental health workforce recruitment and retention across the globe. During this time, mental health providers reported increased acuity of stress, burnout, and high intention to leave, which, ultimately, resulted in costly and unprecedentedly high rates of turnover (Jarden et al., 2023; Poindexter, 2022). However, less examined are the staff members that remained during this time and the elements within their work environment that supported their resilience within this large-scale system shock. Research is needed to illuminate the factors that support workforce retention for future system shocks.

## Assertive Community Treatment

Assertive Community Treatment (ACT) is a cornerstone of public mental health care in the United States. Upwards of 1,100 ACT teams are active in 42 States with three additional States in early stages of implementation (Moser et al., 2024). ACT is a team-based model of care that provides essential health services for people living with psychiatric disabilities who have been ensnared within the ‘institutional circuit’ of housing instability, criminal justice involvement, and/or psychiatric hospitalization (Bond & Drake, 2015; Hopper et al., 1997; Mancini et al., 2009). The essential ingredients of ACT (i.e., transdisciplinary team, active partnership and person-centered care, recovery-oriented, low patient-provider ratios, fixed point of responsibility, in-vivo services, frequency of contact, 24-hour availability, and the shared goal of a meaningful life) guide the identity, purpose, and values around which ACT teams collaborate (Bond et al., 2001; Bond & Drake, 2015). When ACT teams are comprised of less committed or skilled staff, or experience poor intra-team relationships or ineffective leadership, the quality of services (Mancini et al., 2009) and staff retention decrease (Mancini et al., 2009; Zhu et al., 2017).

### High ACT Staff Turnover

Recent data suggests staff retention is one of the most pressing challenges facing ACT teams and ACT team leaders. Among a national sample of ACT team leaders (*N* = 302), 70% reported that recruitment and retention are significantly worse post-pandemic compared to pre-pandemic. The majority of established teams (*N* = 282) had only retained approximately a quarter of the staff on their team for three years or longer (Moser et al., 2024). This means that 75% of the staff on these ACT teams turned over in the last three years marking tremendous disruption for teams. However, this high turnover rate was not present in all teams. Understanding which leadership and supervision strategies are associated with lower voluntary turnover intention among ACT team members provides an opportunity to meet this critical workforce challenge.

## Voluntary Staff Turnover Intention

Turnover intention, or one’s voluntary intention to leave their organization, has been the strongest predictor (*p* =.56) of actual voluntary turnover among individual, job, and organizational characteristics in a meta-analysis of voluntary turnover across all types of organizations (Rubenstein et al., 2018). Similarly, intention to leave was the strongest predictor of voluntary turnover among staff working in child welfare settings (Griffiths et al., 2020). Many factors can push staff members to develop an intention to leave their positions, including high stress and burnout (Beidas et al., 2016; Brabson et al., 2020), job satisfaction (Fukui et al., 2020), work-life conflict and personal non-work-related characteristics, (e.g., parenting children under 5 years old; Rubenstein et al., 2018). Less understood are the malleable levers that *pull* staff members to stay and reduce their intention to leave within the context of large-scale shocks (Waldman et al., 2015).

## Supervisory Working Alliance

Conceptual and empirical scholarship point to supervisory working alliances between supervisors and their supervisees as a key predictor of supervisees’ outcomes, including intention to voluntarily leave their positions (Goodyear, 2014; Kadushin & Harkness, 2002; Mor Barak et al., 2009). The supervisory working alliance has been defined as the degree of supervisor-supervisee bond, agreement on goals, and focus on service user-related outcomes and tasks (Borders et al., 2023; Sabella et al., 2020). The majority of evidence related to the supervisory working alliance has focused on supervision outcomes, such as burnout, job satisfaction, and competence (Sewell et al., 2024) and supervision within training contexts (Borders et al., 2023; Bearman et al., 2017; Ertl et al., 2023). One randomized trial found that supervisory training that strengthened the supervisory working alliances was significantly related to stronger therapeutic alliances between supervisees and their clients and reductions in depression among clients (Bambling et al., 2006). However, less research has focused on actionable guidance for supervisors on how to develop effective supervisory working alliances in the context of public mental health care settings.

## Transformational Leadership

Abundant evidence suggests that transformational leadership behaviors will support supervisee retention (Avolio et al., 2009; Bass, 1999) and potentially supervisory working alliances with staff. Transformational leadership encompasses behaviors of: idealized influence (inspiring others to want to be or act like you), inspirational motivation (appealing to the mission and values of the work), intellectual stimulation (identifying and encouraging pursuit of staff curiosities or gifts to stay engaged), and individualized consideration (attending to those individualized needs and interests) (Aarons, 2006; Bass, 1999). In mental health care settings, transformational leadership has been associated with reduced staff intentions to leave and attenuation of the relationship between intention to leave and turnover behavior (Green et al., 2013), reduced staff burnout (Corrigan et al., 2003) and stress (Fukui et al., 2020), positive influence on staff attitudes (Farahnak et al., 2020), improved quality of life, and satisfaction of people receiving mental health services (Corrigan et al., 2000). Transformational leadership includes behavioral elements (e.g., modeling, charisma, consideration of each staff member, and mission focus) that conceptually align with strong supervisory working alliances, but as of yet, associations between transformational leadership, supervisory working alliance, and team member intention-to-leave remain under-investigated.

## ACT Implementation Leadership

ACT team leaders are primarily responsible for implementing ACT with fidelity to the model as intended (Monroe-DeVita et al., 2011) through their coordination and clinical supervision of an interdisciplinary team. ACT fidelity requires regular clinical supervision for team members and oversight of recovery plans. Despite this integral role, no guidance exists for how team leaders are to effectively enact these responsibilities and which strategies are most critical for them to use. Experts were surveyed in order to identify and rate the most important ACT team leader activities, which included facilitating person-centered care approaches, reviewing person-centered care planning, obtaining feedback from staff and clients, and encouraging constructive feedback within team meetings (Carlson et al., 2012). Leadership in the context of implementation of evidence-based practices like ACT is critical for outcomes (Aarons et al., 2011). However, no evidence exists of whether and how team leaders engage these strategies and if they support the retention of ACT team members.

### Team Cohesion

Team cohesion, defined as the degree to which team members are committed to both their shared tasks and each other, can have a significant influence on the ability of teams to function effectively and to constructively disagree in order to identify the best path forward within their tasks (Kozlowski & Ilgen, 2006). The presence of team cohesion may be particularly important within ACT teams because of a high level of interdependence of their collaborative work with people receiving services. Conversely, team interpersonal conflict and lack of cohesion can be an influential driver of the development of intentions to leave mental health care roles (Yanchus et al., 2017). This negative conflict within teams has been demonstrated to lead to turnover (Zhu et al., 2017).

## Evidence-Based Clinical Supervision Strategies

ACT team leaders often carry both administrative leadership and oversight of clinical care, which is provided primarily through weekly clinical supervision with supervisees. Clinical supervision is education and support for clinicians within the context of a supervisory relationship in order to ensure ethical and competent practice (Kadushin & Harkness, 2002). Clinical supervision is used across the allied mental health professions to train and license clinicians (American Psychological Association, 2014; National Association of Social Workers and Association of Social Work Boards, 2013) and beyond licensure, to provide ongoing opportunities for reflection, support, and learning new practice techniques and skills (Bearman et al., 2017; Martino et al., 2016). Within clinical trials, the integration of active learning strategies, performance review, and feedback within supervision has been associated with supervision and client outcomes (Choy-Brown et al., 2022). Yet, a paucity of research has examined effects of using empirically supported supervision strategies within workplace-based mental health services environments where regular supervision is inconsistent (Choy-Brown & Stanhope, 2018; Dorsey et al., 2018). In addition, less understood is the integration of leadership behaviors and clinical supervision strategies that support performance and retention.

## Study Theoretical Model and Hypotheses

**Fig. 1:**
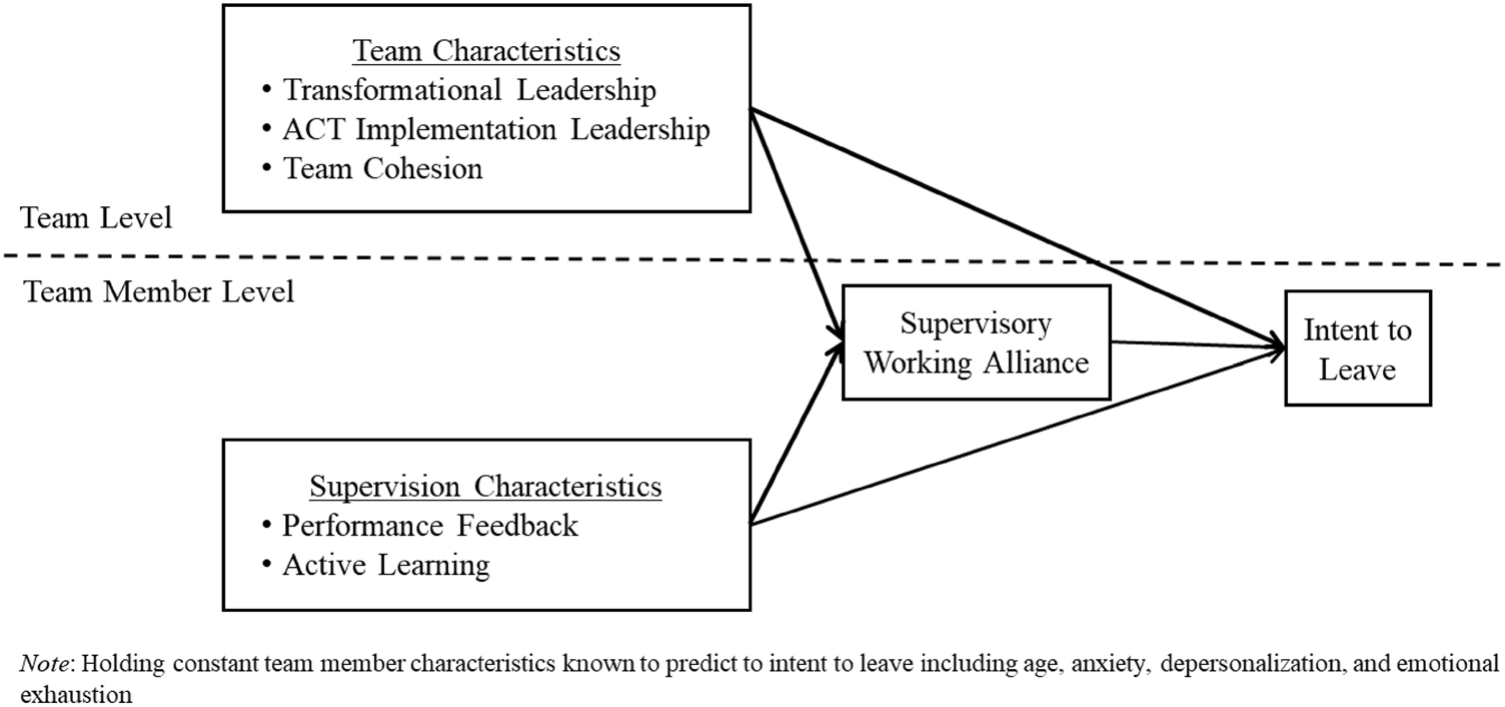
Conceptual model of team and supervision predictors of ACT team member intent to leave

As depicted in the conceptual model (Fig. 1), the present study aims to identify supervisory strategies for disrupting voluntary intention to leave among ACT team members by evaluating team member and team characteristics associated with positive supervisory relationships and reduced voluntary turnover intention. Findings will add to limited literature examining cross-level effects of supervision- and team-level factors on the supervisory working alliance and voluntary turnover intention among ACT teams and may identify potentially actionable supervision strategies to effectively promote workforce retention within public mental health. Specifically, this study evaluates the following hypotheses:

### • Hypothesis 1

• Supervisory working alliance will be negatively associated with supervisees’ intention to leave.

### • Hypothesis 2

• Transformational leadership will be positively associated with supervisory working alliance and negatively associated with supervisees’ intention to leave.

### • Hypothesis 3

• ACT implementation leadership will be positively associated with supervisory working alliance and negatively associated with intention to leave.

### • Hypothesis 4

• Team cohesion will be negatively associated with intention to leave and positively associated with supervisory working alliances.

### • Hypothesis 5

• Evidence-based clinical supervision strategies (use of behavioral rehearsal, modeling, and data informed feedback) will be positively associated with supervisory working alliance and negatively associated with intention to leave.

### Methods

This study used a cross-sectional observational survey design to explore team and supervisory characteristics associated with ACT team members’ intention to leave their positions working on an ACT team amidst the COVID-19 pandemic in 2021.

### Sample

**Fig. 2:**
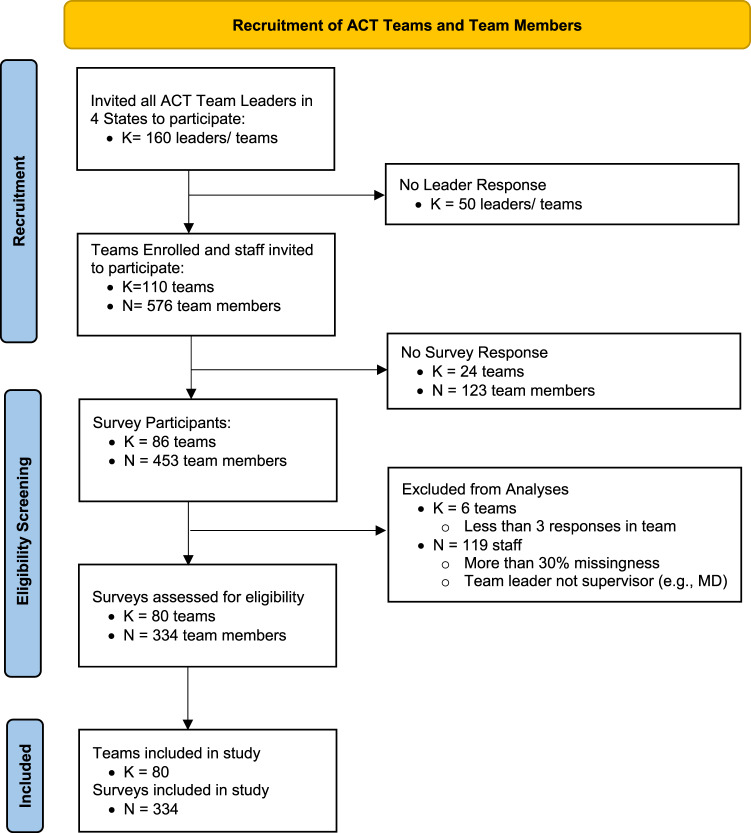
Recruitment and Eligibility Screening Flow of Included Participant Sample

Providers working on ACT teams in four States in the Northwest, Midwest, and Southeastern U.S. were recruited to participate in the study (Fig. 2). The four states were selected for the range in region, number of teams within the state, strength of technical assistance and administrative ACT support, acceptable fidelity ratings across teams, and reimbursement rates. ACT teams were eligible for the study if they were in (1) one of the four targeted States, (2) in good standing, and (3) continued operation during the study. ACT team members were eligible for the study if the ACT team leader was their primary supervisor. Team members included all interdisciplinary providers on ACT teams, including: social workers, nurses, peer specialists, employment specialists, and co-occurring disorder specialists. After excluding providers with *≥* 5% missing survey responses and teams in which fewer than three team members responded, the final analytic sample included 334 providers working in 80 teams with a range of 3 to 20 team members (M = 10.78, SD = 3.19) per team. Based on simulation procedures described by (Bodenheimer & Sinsky, 2014), the sample size of *n* = 334 was adequate to achieve power > 0.8 for all parameters of interest.

### Procedures

 ACT team leaders (*n* = 160*)* working in the four eligible states received an electronic invitation to enroll their teams in the survey; 69% (*n* = 110) of teams enrolled. After a team leader enrolled their team, an email invitation was sent directly to all team members working on the team. Team members responded to an email invitation to participate in the approximately 20-minute web-based survey from May to December 2021. Team members provided electronic informed consent prior to participation and received a $20 electronic gift card. Team members received up to two reminders to complete the survey within a week and then two weeks from the initial email. All procedures were approved by the affiliated Institutional Review Board.

### Measures

#### Outcomes

**Intention to leave.** Intention to leave was operationalized as employees’ self-reported plan to leave their position, consistent with previous research (Albott et al., 2020; Mills et al., 2020). It was measured using the 4-item Intent to Leave Scale (Abrams et al., 1998; Nissly et al., 2017), which assessed intention to leave (e.g., “In the next few months I intend to leave this organization”) on a 5-point Likert-type scale from 1 (“Strongly disagree”) to 5 (“Strongly agree”). A higher score indicates greater intention to leave the organization. Coefficient alpha in this sample was acceptable (α =.78).

**Supervisory working alliance.** The Brief Supervisory Working Alliance Inventory– Trainee Form (BSWAI-T) (Sabella et al., 2020) included items assessing supervisor-supervisee rapport and the client focus (5-items;). Coefficient alpha in this sample was good (α = 0.82). Items were rated on a 7-point Likert-type frequency scale from 1 (“Almost never”) to 7 (“Almost always”). A higher score indicates a greater supervisory working alliance.

### Supervision Characteristics

**Active skill building and performance-based feedback in Supervision.** The Evidence-Based Clinical Supervision Strategies Scale (Choy-Brown et al., 2023) assessed two evidence-based supervision strategies using separate sub-scales of performance-based feedback (3-items; α = 0.82) and active skill building (2-items). Coefficient alpha in this sample was good (α = 0.83). Items were rated on a 5-point Likert-type scale from 1 (“Not at all”) to 5 (“Always”) indicating the frequency with which the supervisee experienced each strategy in supervision. A higher score indicates greater frequency of the experience of these supervision strategies.

### Team Level Characteristics

Team level characteristics are constructs that describe the ACT team as a whole and are supported by empirical evidence of high within-team agreement on construct ratings. Variables that measure team-level constructs were generated by aggregating team members’ individual ratings (i.e., calculating the team mean) when there was justifiable within-team inter-rater agreement, as indicated by *R*_*wg(j)*_ values greater than the recommended cutoff of 0.7 (James et al., 1993; Lengnick-Hall et al., 2023). Including these characteristics as team-level variables is consistent with organizational and leadership studies evaluating associations between team-level transformational leadership (Farahnak et al., 2020), team cohesion (Zhu et al., 2017), and implementation leadership (Aarons et al., 2014) with both team-level and individual-level outcomes.

**Transformational leadership.** The transformational leadership subscale of the Multifactor Leadership Questionnaire (MLQ; Avolio et al., 1999) was used to assesses team members’ perceptions of their supervisors’ transformational leadership behaviors, including: individualized consideration, intellectual stimulation, and idealized influence (behavior and attributed) The subscale includes 20 items rated on a 5-point Likert-type scale from 1 (“Not at all”) to 5 (“If not always”). Coefficient alpha in this sample was high (α = 0.97). Example items included: “Instills pride in me for being associated with him/her/them” and “Articulates compelling vision of the future.” A higher score indicates more frequent use of transformational leadership behaviors by the supervisor, as reported by supervisees.

**Team cohesion.** Five items were drawn from the ACT team constructive conflict climate subscale of the Teamwork in ACT scale (Wholey et al., 2012; Zhu et al., 2017) to examine cohesion (low relationship conflict) among team members. The subscale includes 5 items rated on a 4-point Likert scale from 1 (“Strongly disagree”) to 4 (“Strongly agree”). Coefficient alpha in this sample was α = 81. Example items included: “I felt that on my ACT team we used our opposing views to understand problems” and “I felt that even when we disagreed on my ACT team we communicated with respect for each other.” A higher score indicates great team cohesion and ability to use disagreement to improve teamwork.

**ACT implementation leadership.** This 11-item scale was generated from items that had previously been rated by experts as key leadership behaviors for ACT (Carlson et al., 2012). Participants were asked to rate each ACT team leader behavior on a 5-point Likert-type scale from 1 (“Not at all) to (“If not always”). Example items included: “The Team Leader encourages team members to both give and receive feedback from peers in a positive manner in team meetings” and “The Team Leader uses strategies to facilitate the team members’ use of person-centered behaviors, language and ideas that are consistent with ACT philosophy/principles.” Coefficient alpha in this sample was high (α = 0.94). A higher score on the ACT implementation leadership scale indicates more frequent use of what experts rated as critically important ACT team leader behaviors.

### Team Member Characteristics

**Depersonalization and emotional exhaustion**. Four items from the Maslach Burnout Scale (Maslach et al., 1997) were used to assess the degree to which team members reported depersonalization (2-items, α = 0.67) and emotional exhaustion (2-items). Coefficient alpha in this sample was high (α = 0.91). The items for emotional exhaustion included: “I feel emotionally drained from work” and “I feel used up at the end of the workday.” The two items for depersonalization included: “I have become more callous toward people” and “I don’t really care what happens to some consumers.” These items were rated on a 7-point Likert-type scale from 0 (“Never”) to 6 (“Everyday”). These items were selected from each subscale to reduce participant burden and for their high factor loadings on the construct (Maslach & Jackson, 1981).

**Generalized Anxiety**. The Generalized Anxiety Disorder– 2 subscale (GAD2) (Kroenke et al., 2007) from the Generalized Anxiety Disorder- 7 scale (Spitzer et al., 2006) was used to assess general anxiety among team members within the last two weeks. The coefficient alpha in this sample was good (α = 0.85). Items are rated on a 4-point Likert-type scale from 0 (“Not at all”) to 3 (“Nearly every day”). The two items included: “Over the last two weeks, how often have you been bothered by feeling nervous, anxious, or on edge” and “Over the last two weeks, how often have you been bothered by not being able to stop or control worrying.” A higher score indicates higher severity of anxiety.

Preliminary analyses indicated other team member demographic variables were not significantly correlated with the outcome variables and therefore were not included.

### Data Analysis

Hierarchical linear models, sometimes called mixed models, were the general analytic approach because they permit the inclusion of predictors at multiple levels (i.e., team member, supervisor, team) and account for the clustering of team members within ACT teams via random intercepts (Raudenbush & Bryk, 2002). Models were estimated using restricted maximum likelihood via the mixed command in Stata 18.0. Missing data (< 5%) were addressed through multiple imputation (20 data sets) using the suite of mi estimate commands in Stata 18.0. (*StataCorp*, 2021).

To answer the research questions, four models were estimated. First, bivariate models were used to estimate the unadjusted relationships between each predictor and our focal outcome of intention to leave. Second, bivariate models were used to estimate the unadjusted relationships between each predictor and our secondary outcome of supervisory working alliance. Third, the adjusted relationships between the full set of predictors and supervisory working alliance were examined. Fourth, the adjusted relationships between the full set of predictors and our primary outcome, intention to leave, were examined. Structuring our analyses in this way allowed us to identify the individual relationships between predictors at each level and outcomes as well as the significant relationships when holding constant all other variables. To provide a measure of effect size, we calculated an analogue to Cohen d for each unadjusted and adjusted relationship between predictor and outcome. The Cohen d value was calculated as the standardized marginal mean difference in the outcome, contrasting groups +/- 1 standard deviation from the mean [*d* = (*M*_2 adj_ - *M*_1 adj_) ⁄ *SD*_pooled_], where *M*_2 adj_ = the marginal adjusted mean on the outcome for a group + 1 standard deviation above the mean on the predictor, *M*_1 adj_ = the marginal adjusted mean on the outcome for a group − 1 standard deviation below the mean on the predictor, and *SD*_pooled_ = the standard deviation of the outcome variable (Cohen, 1988). The conventions for interpreting Cohen’s d are typically 0.2 is small, 0.5 is medium, and 0.8 is large (Cohen, 1988).

## Results

### Sample Characteristics

Team members (*N* = 334) were working to provide services in *K* = 80 ACT teams across four states (see Table 1). This represents an average within team provider response rate of 50% among the teams that enrolled (K = 110) (Fig. 2). The demographic characteristics of participating ACT team members were consistent with what one might expect for the workforce across the four states. On average, team members had been working on their team for 4.7 years (SD = 4.9) and with their team leader for 2.6 years (SD = 3.1) (Table 1). Descriptive statistics of the team, supervision, and team member characteristics are listed in Table 1. Notably, the mean reported sense of depersonalization was between “never” and “a few times a year or less” (M = 1.9, SD = 2.4, out of 7), indicating that on average ACT team members report infrequent experiences of callousness and disregard for people seeking services, which is consistent with the values and approach of ACT.

**Table 1:** Sample demographic characteristics and descriptive statistics (*N* = 334)

	M	SD	Min	Max	Missing (%)
**Team Characteristics (K = 80)**					
ACT Implementation Leadership	2.55	0.98	0	4	3
Team Cohesion	3.07	0.69	1	4	2
Transformational Leadership	3.04	0.86	0	4	5
Team Size	10.78	3.19	3	20	0
**Supervision Characteristics (*n*** **= 334)**					
Supervisory Alliance	5.7	1.56	1	7	2
Active Learning Strategies	2.67	1.2	1	5	< 1
Feedback Strategies	3.01	1.15	1	5	2
Hours of Supervision	4.84	3.59	0	24	4
**Team Member Characteristics (*n*** **= 334)**					
Intent to Leave	9.8	4.2	4	20	< 1
Age (years)	43.3	12.1	21	77	10
GAD-2	0.73	0.83	0	3	< 1
Years within the Organization	4.7	4.9	0	30	< 1
Years working with the Team Leader	2.6	3.1	0	28	3
Depersonalization	1.9	2.4	0	12	2
Emotional Exhaustion	6.5	3.2	0	12	< 1
	***n***	***%***			
Gender Identity					0
Woman	274	82			
Man	51	15.3			
Non-Binary	4	1.2			
Prefer to Not Respond	4	1.2			
Transgender	1	0.3			
Self-Describe	1	0.3			
Racialized Identity					0
White	268	80.2			
Black	36	10.8			
Prefer to Not Respond	12	3.59			
Self-Describe	8	2.4			
American Indian/Alaska Native	6	1.8			
Asian	6	1.8			
Hispanic or Latine	14	4.2			0
Lived Experience of Serious Mental Illness	43	12.9			0
Highest Level of Education					< 1
Bachelor’s Degree	135	40.7			
Master’s Degree	92	27.7			
Associates Degree	55	16.6			
High School Degree	27	8.1			
Other	18	5.4			
Doctoral or Medical Degree	5	1.5			
Role on the Team					0
Nurse	82	24.6			
Clinician/Mental Health Professional	49	14.7			
Care Coordinator	43	12.9			
Vocational Specialist	35	10.5			
Substance Abuse Specialist	32	9.6			
Peer Specialist	31	9.3			
Program Assistant	28	8.4			
Assistant Team Leader	12	3.6			
Housing Specialist	9	2.7			
Psychiatric Care Provider	6	1.8			
Other	6	1.8			
Type of Employment					0
Salaried	182	54.5			
Hourly/Independent Contractor	152	45.5			

### Predictors of ACT Team Members’ Intention to Leave

Table 2 presents unadjusted and adjusted parameter estimates at the team member, supervision, and team level from the hierarchical linear models predicting the ACT team members’ intention to leave.

*Team member characteristics.* In the unadjusted bivariate models, four team member characteristics were significantly associated with increased intention to leave, including: higher depersonalization (d = 0.76), higher emotional exhaustion (d = 0.73), and higher generalized anxiety (d = 0.63). Age was significantly associated with reduced intention to leave (d=−0.42), In the adjusted model, all four team member factors remained significantly positively associated with intention to leave, including: higher depersonalization (d = 0.31), higher emotional exhaustion (d = 0.38), and higher generalized anxiety (d = 0.21). Team member age was significantly negatively associated with intention to leave (d=−0.24 (Table 2).

**Table 2:** Predictors of ACT team members’ (*N* = 334) intent to leave in unadjusted and adjusted models

	Unadjusted				Adjusted^1^			
	b	SE	CI -/+		b	SE	CI -/+	
Team Member Characteristics								
Age (years)	−0.02**	0.00	−0.03	−0.01	**−0.01**	0.00	−0.02	0.00
Depersonalization	0.38**	0.05	0.28	0.48	0.16*	0.06	0.04	0.27
Emotional Exhaustion	0.37**	0.05	0.27	0.47	0.20**	0.06	0.08	0.31
GAD-2	0.32**	0.05	0.22	0.42	**0.11**	0.05	−0.00	0.21
Supervision Characteristics								
Supervisory Alliance	−0.30**	0.05	−0.41	−0.20	−0.16*	0.06	−0.27	−0.05
Supervision Active learning	−0.15*	0.05	−0.25	−0.04	−0.03	0.06	−0.15	0.10
Supervision Feedback	−0.21**	0.05	−0.32	−0.11	−0.04	0.06	−0.16	0.09
Team Level Characteristics								
ACT Implementation Leadership	−0.25**	0.06	−0.37	−0.12	0.03	0.11	−0.20	0.25
Team Cohesion	−0.19**	0.06	−0.31	−0.06	−0.05	0.06	−0.17	0.07
Transformational leadership	−0.26**	0.07	−0.39	−0.13	−0.16	0.12	−0.39	0.07

Age coefficient is not standardized. Other coefficients are standardized

^1^Adjusted model included covariate for state

Bold = significant at *p*<.05, * *p*<.01, **p<.001

*Supervision characteristics.* In the unadjusted models, all supervision characteristics were significantly associated with decreased intention to leave. Lower ratings of the supervisory working alliance (d=−0.62) and lower use of active learning (d=−0.29) and performance-based feedback (d=−0.43) were all significantly associated with increased intention to leave in the unadjusted models. In the adjusted model, the supervisory working alliance was significantly associated with intention to leave such that higher supervisory working alliance was associated with reduced intention to leave (d=−0.32).

*Team characteristics*. In the unadjusted models, higher use of ACT team leadership (d=−0.50), higher transformational leadership by the ACT supervisor (d=−0.52), and higher team cohesion (d=−0.38) were all significantly associated with reduced intention to leave. In the adjusted model, higher transformational leadership was the only team characteristic that was significantly associated with reduced intention to leave (d=−0.33).

#### Predictors of ACT Team Members’ Supervisory Working Alliance

*Team member characteristics.* In the unadjusted bivariate models, decreased depersonalization (d=−0.58) and emotional exhaustion (d=−0.38) were significantly associated with higher supervisory working alliance. In the adjusted model (Table 3), only decreased depersonalization was significantly associated with higher supervisory working alliance (d=−0.41). Age and general anxiety were not significantly associated with supervisory working alliance in either the unadjusted or adjusted models.

*Supervision characteristics.* In the unadjusted models and the adjusted model (Table 3), both higher frequency of active learning (d = 0.83 and d = 0.18) and performance feedback (d = 0.90 and d = 0.48) during supervision time were significantly associated with higher supervisory working alliance.

*Team characteristics*. In the unadjusted models, all team characteristics including higher team cohesion (d = 0.61), ACT implementation leadership (d = 0.93), and transformational leadership of the ACT team leader (d = 0.98) were significantly associated with higher reported supervisory working alliance (Table 3). In the adjusted model, higher team leader transformational leadership was significantly associated with higher supervisory working alliance (d = 0.75).

**Table 3:** Predictors of ACT team members’ (*N* = 334) supervisory working alliance with their ACT team leader (*N* = 80) in unadjusted and adjusted models

	Unadjusted				Adjusted^1^			
	b	SE	CI -/+		b	SE	CI -/+	
Team Member Characteristics								
Age (in years)	0.00	0.00	−0.01	0.01	−0.00	0.00	−0.01	0.01
Depersonalization	−0.29**	0.05	−0.39	−0.18	−0.20**	0.05	−0.31	−0.10
Emotional Exhaustion	−0.19**	0.05	−0.30	−0.09	−0.01	0.06	−0.12	0.10
GAD-2	−0.09	0.05	−0.19	0.02	0.03	0.05	−0.07	0.13
Supervision Characteristics								
Supervision Active learning	0.41**	0.05	0.31	0.51	0.18*	0.06	0.06	0.29
Supervision Feedback	0.45**	0.05	0.35	0.55	0.24**	0.06	0.12	0.36
Team Level Characteristics								
ACT Implementation Leadership	0.47**	0.06	0.35	0.58	−0.05	0.11	−0.27	0.16
Team Cohesion	0.31**	0.06	0.18	0.43	−0.04	0.06	−0.16	0.08
Transformational leadership	0.49**	0.06	0.38	0.61	0.38**	0.11	0.16	0.59

Note: Age coefficient is not standardized. Other coefficients are standardized

^1^Adjusted model included covariate for state

Bold = significant at *p* <.05, * *p* <.01, **p <.001

## Discussion

Findings provide supportive evidence for actionable and malleable supervisory strategies that ACT team leaders can employ to reduce team members’ intention to leave their positions within large-scale shocks. Beyond team characteristics, stronger supervisory relationships between ACT team members and their supervisor were negatively associated with intention to leave even within a context of emotional exhaustion and anxiety. While ACT implementation leadership, transformational leadership, and team cohesion were significantly negatively correlated with intention to leave in the bivariate models, once other leadership, supervisory, and team member characteristics were held constant, the effect was no longer significant. As such, the critical importance of building alliances with supervisees, including treating staff members as colleagues and focusing on client process and outcomes have evidence of reducing team members’ intention to leave the team.

In turn, team leaders’ transformational leadership was positively associated with stronger supervisory working alliances. While a formal test of mediation was beyond the scope of these data due to the absence of longitudinal data, the pattern of relationships observed is consistent with a mediation model in which the supervisory relationship plays a role in mediating the link between ACT team leader leadership style and ACT team member retention. This potentially mediating role of the supervisory relationship adds to the evidence supporting the negative association between transformational leadership and intention to leave (Green et al., 2013; Park & Pierce, 2020). Such leadership behaviors are modifiable and interventions to improve transformational leadership behaviors among mental health and substance use providers have been successful in clinical trials (Aarons et al., 2017; Williams et al., 2024). Prevention of staff turnover likely requires the creation of work environments that promote staff resilience to external system shocks, and these malleable supervisory behaviors have evidence of effectiveness (Jackson et al., 2021).

Beyond just foundational emotional support, a novel finding in this study is that supervisees whose supervisors successfully translated their expertise through evidence-based supervision strategies of behavioral rehearsal and feedback reported significantly stronger supervisory working alliances and lower turnover intentions. The transfer of expertise may make workers more able to respond with resilience to the consistent demands of community mental health service provision and disruptive large-scale external system shocks (Williams & Beidas, 2018). This suggests that supporting learning within supervision is both a tool for supervisees’ clinical knowledge and skill development *and* for the strengthening of a protective supervisory relationship. By creating a relationship that includes active learning strategies– i.e., rehearsing and modeling practice techniques– supervisors can provide supervisees clarity about both what to do and how to practice. The use of behavioral rehearsal strategies during supervision time can also provide the supervisor with an opportunity for direct observation that has been shown to approximate quality of supervisees’ clinical interactions (Becker-Haimes et al., 2022; Beidas et al., 2014). This provides an opportunity for performance-based feedback– another empirically supported supervision intervention (Dorsey et al., 2018). This finding clarifies supervisory behaviors that pull employees to stay in their roles, rather than collapsing supervisory behaviors, alliance, and leadership into a single supervisor-rated factor (McFadden et al., 2015). These findings also add to the existing evidence to support the adage that people leave supervisors not jobs.

In the adjusted model predicting voluntary intention to leave, in addition to supervisory working alliance, the team member characteristics of age, emotional exhaustion, depersonalization, and generalized anxiety were also important. Older team members had lower intention to leave in this sample, which is consistent with previous research about younger team members perhaps moving on for professional growth (Fukui et al., 2020; Rubenstein et al., 2018). In the context of working in an essential health care role during the COVID-19 pandemic, the association of anxiety and intention to leave may have been inflated, given the prevalence of anxiety among health care workers in general due to increased safety and health concerns (Amsalem et al., 2023). The associations between higher emotional exhaustion, depersonalization, and intention to leave are consistent with the evidence that burnout plays a significant role in driving up intention to leave among the community mental health workforce (Paris & Hoge, 2010) and reduced workforce turnover among healthcare professionals (Lee et al., 2021; Maniscalco et al., 2024). However, the sample mean for depersonalization was low (Table 1) and indicates that despite the external stressors of the time, team members on average nearly always maintained their consideration of the humanity of those that they served.

In the adjusted model predicting supervisory working alliance, in addition to transformational leadership and evidence-based clinical supervision strategies, team members’ depersonalization was negatively associated with supervisory working alliance. While depersonalization remained rare on average, this negative association points to a potentially critical opportunity to reduce depersonalization of service users through actionable supervisory strategies, including strengthening supervisory working alliance through modeling and rehearsal of practice interactions, direct observation of supervisees’ practice, and informed feedback within the context of supervision interactions. This finding is also consistent with previous evidence of associations between supervision and reduced burnout (O’Connor et al., 2018).

Identifying predictors of reduced depersonalization is important, particularly for team members working with people with psychiatric disabilities for which person-centered care approaches are central for successful service provider-service user interactions (Atterbury, 2014; Stanhope et al., 2013, 2021; Tondora et al., 2014). In the context of ACT services, team members’ depersonalization of service users directly threatens the team’s core values and is antithetical to the ethical imperative of recovery-oriented care. Recovery-oriented care centers hope, humanity, personhood, and self-determination and is a required component within ACT services (Bond & Drake, 2015; Davidson et al., 2007; Monroe-DeVita et al., 2012). This adds quantitative evidence of the potential of supervision strategies to improve the delivery of person-centered, recovery oriented care to existing qualitative evidence (Choy-Brown, 2021). Consequently, supervision-focused interventions supporting team leaders to engage in these supervisory leadership and supervision strategies may improve both team member experiences, retention, and promote positive engagement in the delivery of high quality, recovery-oriented ACT care.

### Strengths & Limitations

These study findings must be considered within the context of the study’s strengths and limitations. This study includes data from the largest survey of ACT team members working in the United States and ACT teams known to these authors. Furthermore, it addresses ACT team members and leaders during the global COVID-19 pandemic, when burnout and turnover became heightened challenges to the mental health work force. Incomplete response from participating States’ team leaders and participating team members may introduce response bias that constrains the impact of findings. However, robust analytic techniques were used to account for missing data. The cross-sectional data prevents us from making causal attributions about the relationships observed in this study and constrained our ability to evaluate supervisory working alliance as a mediator of the relationship between team level factors of transformational leadership and intention to leave. Measurement error may have impacted study findings. In particular, the full set of items from the validated scales were not used for depersonalization and emotional exhaustion, which may have introduced measurement error. Measuring turnover behavior, compensation, team leader tenure, and opportunities for promotion may have strengthened our understanding of retention, though this was outside the scope of this study. The large number of statistical tests conducted in this study increases the familywise error rate and with it, the probability of making a Type I error; consequently, it will be important to replicate findings from this study in future research. These findings should be considered in the context that they were collected - a year of exceptional contextual change due to the COVID-19 pandemic (e.g., various stages of community in-person activities in each State, expansion of Medicaid reimbursement for telehealth). ACT teams remained essential and continued to deliver some services in-person and integrated some telehealth service delivery. However, team members response to the changing landscape and varying levels of comfort with the requirements is a potential factor influencing intention to leave. Finally, this study contributes to limited evidence of cross-level leadership and supervisory predictors of team member level outcomes, particularly among Assertive Community Treatment teams. Evidence of team level predictors of team member outcomes can guide supervision-focused interventions with broad impact across teams.

### Implications for Practice and Policy

Supervision and leadership strategies have a significant influence on factors that are critical for team resilience, staff retention, and patient care. Shifting our focus to investment in existing human resources illuminates a potential supervisory-focused pathway to further promote resilience for essential workers. Actionable supervision strategies (e.g., building rapport, skill building, intellectual stimulation, individualized consideration) may improve staff retention & improve consideration of the humanity of clients– a critical ingredient for the quality of care.

Leveraging under-utilized supervision time to expose staff to empirically supported supervisory behaviors may have critical implications for recruiting and retaining a competent, engaged, and motivated workforce and improving the quality of care for people living with serious mental illness. Policy makers can use these findings to guide focused investments to promote the growth and expertise of this critically important supervisory workforce segment. These findings fill a gap in our understanding of actionable strategies ACT team supervisors can use to fortify their teams and supervisees against external shocks that contribute to voluntary turnover while providing essential healthcare to people living with serious mental illness.
